# An Analysis of the Readability of Public-Facing Information Relating to Prevention of Infectious Diseases by Vaccination

**DOI:** 10.3389/bjbs.2025.15435

**Published:** 2025-12-22

**Authors:** Beverley C. Millar, Callum Peters, John E. Moore

**Affiliations:** 1 School of Biomedical Sciences, Ulster University, Coleraine, Northern Ireland, United Kingdom; 2 Department of Bacteriology, Northern Ireland Public Health Laboratory, Belfast City Hospital, Belfast, Northern Ireland, United Kingdom

**Keywords:** immunisation, lay stakeholders, lay writing, readability, scientific communication, infectious diseases, vaccine, vaccination

## Abstract

**Purpose:**

The readability of public-facing vaccine-related information is an important aspect of health literacy particularly regarding vaccine uptake. The aims of this study were to analyse the readability of such written literature and to provide recommendations, for improvement.

**Methods:**

Readability of vaccine-related information (n_total_ = 240) from publicly available sources (n = 20 per category), including PubMed Abstracts, Expert Review of Vaccines (ERV) and Cochrane Reviews (CR), paired plain language and scientific abstracts, public health materials, clinical trial summaries and vaccine patient information leaflets, were assessed using the Flesch Reading Ease (FRE), Flesch-Kincaid Grade Level (FKGL), SMOG and Gunning Fog readability metrics using the readability software tool readable.com.

**Results:**

Vaccine-related information for all sources had poor readability across all readability metrics with 90.8% and 94.6% not reaching the target FKGL (≤8) (mean 12 ± 3.2 sd) and FRE (≥60) (mean 34 ± 17 sd). Plain language summaries had improved readability, but did not reach reference targets. Scientific abstract and plain language scores for the CR were FRE (mean 25 ± 7.2 sd; median 25) versus (mean 37 ± 8.6 sd; median 36) p < 0.0001), respectively and for ERV FRE the scientific abstract (mean 18 ± 11 sd; median 17) versus the plain language score (mean 26 ± 11 sd; median 28) p = 0.002), respectively, indicating an improvement in readability scores for plain language summaries but again not reaching reference targets.

**Conclusion:**

The readability of public-facing vaccination materials is currently not optimum. The readability can be improved through the employment of readability calculators and ensuring, where possible, the use of mono-syllable words and less than fourteen words per sentence. The preparation of public-facing materials with improved readability scores will help aid in the promotion of health literacy and in turn promote vaccination uptake.

## Introduction

“*The Immunization Agenda 2030: A Global Strategy to Leave No One Behind*” (IA2030) which was endorsed in August 2020, by the 37th World Health Assembly has a primary vision of equality leading to “*a world where everyone, everywhere, at every age fully benefits from vaccines for good health and wellbeing*” [1]. Central to actioning this global strategy is the importance of co-ownership and co-accountability to ensure that all countries have opportunities to implement an effective and comprehensive vaccination programme. Within the World Health Organization documentation “*AI2030 Framework for Action*” there are four key elements namely (i) coordinated operational planning, (ii) monitoring and evaluation, (iii) ownership and accountability and (iv) communication and advocacy [2]. As the success of any immunization agenda is dependent on various stakeholder involvement from both the healthcare and non-healthcare sectors alike, it is important that evidence-based scientific information is available and understandable for informed choices to be made, whether that be at a policy level or individual level.

Of concern, is the fact that in the UK vaccine uptake rates have been declining since 2013–14 [3, 4]. The recent emergence of measles evidences the impact of decreased vaccine coverage in all regions of the UK. The 2024–25 data from England reported that the Mumps, Measles and Rubella (MMR) vaccine coverage for the two doses, MMR1 and MMR2 was 91.8% and 83.7%, respectively at 5 years of age [5]. Furthermore, none of the routine childhood vaccines monitored reached a coverage above the 95% target in the UK [5]. Globally, in addition to measles, other re-emerging infectious diseases such as Pertussis has been attributed to the declining vaccination rates [6]. Several suggestions for the decrease in vaccination, some of which are debated, include vaccine fatigue following the COVID-19 pandemic, vaccine hesitancy, decreasing confidence, lack of information, lack of awareness in the case of absence of diseases which have been minimised due to vaccination, social deprivation, high population mobility attributing to difficulties with access to vaccines and reduction in available funding of vaccination programmes [3, 7].

**FIGURE 1:**
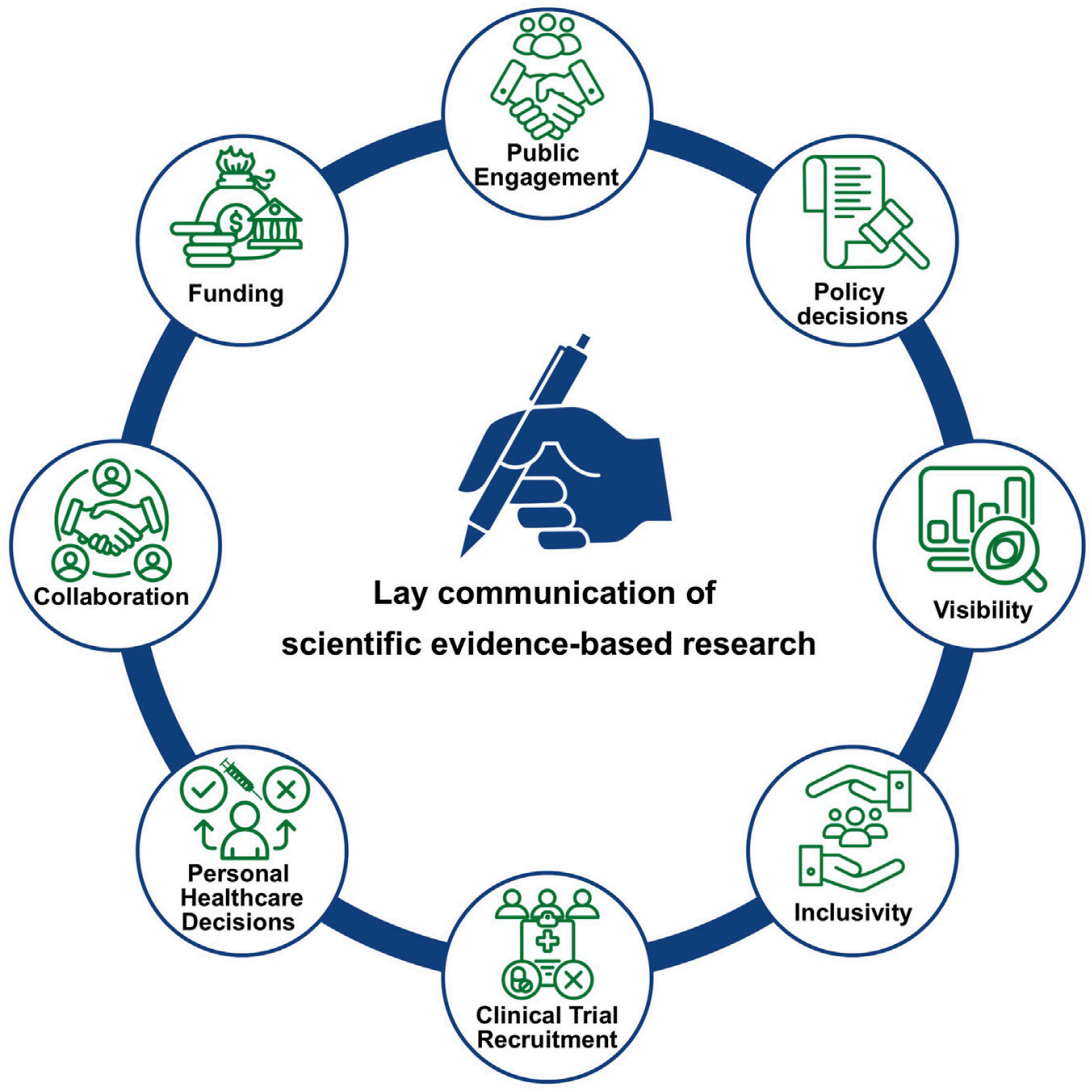
Areas where lay communication of scientific evidence-based research is important.

Community engagement is important to help promote vaccination awareness. Communication of robust scientifically accurate information is important to facilitate trust and alleviate concerns resulting from misinformation. As such, all scientists, particularly those working in healthcare and vaccination, have an obligation to develop their competencies relating to communication with individuals with different levels of scientific understanding (see Figure 1). Such communication is important in relation to the continuation of valuable research through collaboration and funding opportunities as well as ensuring inclusivity and providing evidence-based research findings. This will enable governmental bodies and the public to make informed decisions relating to healthcare delivery.

An important aspect to the formulation of vaccination policies is the scientific literature surrounding the development of novel or improved vaccines and associated clinical trial outcomes, which lead to effective and efficacious vaccination schemes. Historically, such scientific and clinical research findings have been largely reserved for the scientific community, in hard copy subscription-based scientific journals. More recently, the development and availability of the internet have been important factors in facilitating the ability of researchers to search, retrieve and disseminate research more efficiently online. Although hard copies of journals and online journals have provided excellent routes of scientific communication, many journals still continue to require, that either the authors or the readers provide fees to either publish or acquire such articles. The research community has seen further developments in relation to “*open science*,” the aims of which as described by the National Academies of Sciences (2018) are “*to ensure the free availability and usability of scholarly publications, the data that result from scholarly research, and the methodologies, including code or algorithms, that were used to generate those data*” [8]. Many research funders are now promoting or mandating that the outcomes of research studies are published in an open access format. This is highlighted in the United States, where the government promotes public access to results of federally funded research [8]. Open access publishing of research outcomes is beneficial to the general public, thereby aiding with patients’ health literacy. The majority of “open access journals,” however are only free to view, but not free to the authors to publish, with varying associated publication costs. This may translate into authors with limited resources not being able to publish in open access journals thereby potentially depriving the lay audience of full access to relevant scientific studies. Open access publishing of research outcomes is not only available to the scientific community but also the general public, which can be advantageous in relation to areas such as health improvement, however this is commensurate with level of understanding.

Readability is defined as “*a measure of how easy a piece of text is to read. It can include elements of complexity, familiarity, legibility and typography”* [9]. It is central to how well a piece of text can be understood by an individual, particularly those with no specialist knowledge in a subject area. Readability is important as it is linked with health literacy which is defined as “*the capacity of an individual to obtain, interpret and understand basic health information and services in ways that are health-enhancing*” [10]. Low health literacy has been associated with poor health outcomes resulting in more frequent hospital admissions and requirement for emergency care as well as a decrease in the use of various preventative healthcare services such as breast cancer mammogram screening and vaccination uptake [11]. The concept of increased health literacy has a positive correlation with respect to the promotion of vaccine uptake when facilitated by educational interventions [12]. In a meta-analysis by Zhou and colleagues, educational interventions including employment of SMS educational messaging and personal letters were methods that significantly improved vaccine uptake. The educational content of such information focused on the safety and effectiveness of the influenza vaccine, particularly for children and older people, as well as benefits for pregnant women [12]. A study by Plavén-Sigray and colleagues analysed the readability of 709,577 abstracts published in English between 1881 and 2015 from 123 biomedical and life science journals, and observed a decreasing trend in readability on a yearly basis [13]. The authors of this study provided two explanations as to what factors were responsible for this downward trend, namely (i) increased use of scientific jargon and (ii) the cumulative growth of scientific knowledge makes an increasingly complex language necessary [13]. Such a decrease in readability can impact on the accessibility of research materials to both peers, healthcare professionals from other specialisms and the lay community. Scientific journals, such as the Taylor and Francis journal *Expert Review of Vaccines,* have recognised the importance of conveying the research findings relating to vaccines to the lay community and as such, encourage authors of scientific manuscripts to submit plain language summaries [14]. The free availability of such lay information provides a platform to promote public engagement and an understanding of the importance of such scientific research.

The readability of public facing information in numerous healthcare disciplines has been examined; however in relation to vaccine information, the primary focus has been in relation to the COVID-19 pandemic [15–17]. Furthermore, to our knowledge, this is the first study examining the readability of plain language summaries of vaccination focused research articles published in the peer-reviewed literature.

The aims of this study were therefore, to analyse the readability of vaccine related information in written text format from various publicly available sources such as scientific journals, including *Expert Review of Vaccines* and *Cochrane Reviews*, public health materials, clinical trial summaries and vaccine patient information leaflets and to provide recommendations for the improvement of such written literature in order to enhance the understanding of the wider community.

## Methods

### Sources of Information

A cross section of various on-line sources which were freely available and in the public domain, as detailed below, were searched for vaccine-related literature (n_total_ = 240).

### Cochrane Database

Cochrane Reviews (n = 20) published in the Cochrane Library and available from (https://www.cochranelibrary.com/) were obtained having searched the Cochrane Database of Systematic Reviews (CDSR) for articles having the term “*vaccine*” in the title or abstract. Both the scientific abstract and paired plain language summary for the Cochrane Reviews were selected for readability analyses. The Cochrane Central Register of Controlled Trials was searched using the term “*vaccine*” in the title or abstract and articles with vaccine-related information was retrieved (n = 20).

### Pfizer

Two sources of information relating to vaccines were examined from the Pfizer online resource namely, (i) Pfizer plain language study results summaries (n = 20) (available at https://www.pfizer.com/science/clinical-trials/plain-language-study-results-summaries), which provide lay written summaries of clinical trials which were sponsored by Pfizer and for which the target audiences were patients, healthcare providers, caregivers, researchers and the general public and (ii) Pfizer News/Press releases relating to vaccines (n = 20) (available at https://www.pfizer.com/newsroom).

### Public Health Information

A selection of public health information relating to vaccines and vaccination (n = 20), freely available online was sourced from reputable institutions including The European Centre for Disease Prevention and Control (ECDC) (https://www.ecdc.europa.eu/en), the National Health Service UK (NHS UK) (https://www.nhs.uk/), the World Health Organization (https://www.who.int/), the US Department of Health and Human Services (https://www.hhs.gov/immunization/index.html and the US Centers for Disease Control and Prevention (CDC) (https://www.cdc.gov/).

### News Room/Tabloid Articles

Lay information relating to vaccines (n = 20) as distributed by online news broadcasting companies, such as the British Broadcasting Corporation (BBC News), Cable News Network (CNN), Sky News and Fox News, as well as newspapers/tabloids e.g. The Independent, the Guardian, Daily Mail, The Sun, New York Times, and Which, were collected for readability analyses.

### Vaccine Patient Information Leaflets

Patient Information leaflets (PILs; n = 20) accompanying vaccines were sourced from the Electronic Medicines Compendium (https://www.medicines.org.uk/emc).

### Peer-Reviewed Journals

Scientific abstracts (n = 20) relating to the peer-reviewed scientific literature, were selected using PubMed (https://pubmed.ncbi.nlm.nih.gov/), by searching for articles using keywords “*vaccine*,” “*vaccination*,” “*vaccine review”* and named infectious diseases. A scientific journal focusing on vaccines, namely *Expert Review of Vaccines*, was chosen as it permitted the inclusion of a plain language summary alongside its scientific abstract. Articles, published in the years 2020–2024, were searched for and retrieved using the term “*plain language summary.*” The readability of the scientific abstracts (n = 30) and paired plain language summaries (n = 30), were analysed.

### Analysis of Readability of Written Materials

All retrieved information was analysed using the online subscription-based software, Readable (www.readable.com), which was chosen as it had been previously shown to be accurate, permit the input of various formats of written materials and is easy-to-use [18]. The retrieved information was entered into the Readable software in the format of (i) text in the case of PubMed Abstracts, Cochrane Reviews, both plain language summaries and abstracts, Cochrane trials, ERV scientific abstracts and plain language summaries (PLSs), (ii) a pdf file in the case of Pfizer plain language study results and vaccine PILs and (iii) a URL in the case of Pfizer News articles, Public Health information and News/Tabloid information, and analysed as per the software instructions of use.

Four readability indices, namely, the Flesch Kincaid Grade level (FKGL), the Gunning Fog Index, the Simple Measure of Gobbledygook (SMOG) Index and Flesch Reading Ease (FRE) and two text-based metrics namely, words per sentence and syllables per word, were analysed to examine the readability of vaccine-related information. The indices above were chosen as they are the most commonly used readability indices to analyse written healthcare information, particularly FKGL and the FRE [19] and each of the formulae associated with these metrics have specific biases and analyse text differently [20]. Of interest is that the SMOG Index has been proposed as the most suitable and reliable for the analysis of written healthcare information, as it has been validated against one hundred percent comprehension [19]. Additionally, we included two text metrics, namely words per sentence and syllable count, as these have a major influence on readability scores. The formulae associated with each readability index is shown in Table 1.

**TABLE 1:** Readability Formulae used in this study as per Readable.com software.

Readability metric	Formula
Flesch Reading Ease (FRE) US target score: ≥60	$206.835-1.015\;\left(\frac{\text{total}\;\text{words}}{\text{total}\;\text{sentences}}\right)-84.6\;\left(\frac{\text{total}\;\text{syllables}}{\text{total}\;\text{words}}\right)$

Flesch-Kincaid Grade Level (FKGL) *Target Score for general public*: ≤8 8th grade in US school system (13 years of age)	$0.39\;\left(\frac{\text{total}\;\text{words}}{\text{total}\;\text{sentences}}\right)+11.8\;\left(\frac{\text{total}\;\text{syllables}}{\text{total}\;\text{words}}\right)-15.59$

SMOG Index *Target Score for general public*: ≤8 8th grade in US school system (13 years of age)	$3+\sqrt{\text{polysyllabic }}\text{count}$ Calculation details • Count ten sentences near the beginning of the text, 10 in the middle and ten near the end, totalling 30 sentences • Count every word with three or more syllables • Square-root the number and round it to the nearest 10 • Add three to this figure
Gunning Fog Index (GFI) *Target Score for general public*: ≤8 8th grade in US school system (13 years of age)	1.4 $\times \left[\left(\frac{\text{total}\;\text{words}}{\text{total}\;\text{sentences}}\right)+100\;\left(\frac{\text{complex}\;\text{words}}{\text{total}\;\text{words}}\right)\right]$ N.B. Complex words are classed as words with three or more syllables

Adapted from “Readability formulae employed in this study” by Caoimhe Shannon, Beverley C. Millar and John E. Moore, licensed under CC BY 4.0.

### Statistical Analyses

Readability indices and text metric scores, generated by readable.com, for each source of information were analysed statistically, using GraphPad Prism for Windows, Version 10.3.0.507 (GraphPad Software, Boston, USA). Initially a Kolmogorov-Smirnov test for normality was performed, subsequently for lay information data with a normal distribution, a one-way ANOVA with a Tukey’s multiple comparisons test was performed. For data which was not normally distributed, a Kruskal-Wallis test with a *post hoc* by Dunn’s multiple comparisons test was performed. Readability data relating to scientific information namely paired Scientific and Plain Language Summaries from the *Expert Review of Vaccines*, and *Cochrane Reviews* were analysed using a paired student’s t-test. Additionally, scientific information readability values which were normally distributed were statistically compared to PubMed abstract data by means of a one-way ANOVA with a Dunnett’s multiple comparisons test. Statistical significance was set at p ≤ 0.05.

## Results

### Analysis of Readability of Written Materials

**FIGURE 2:**
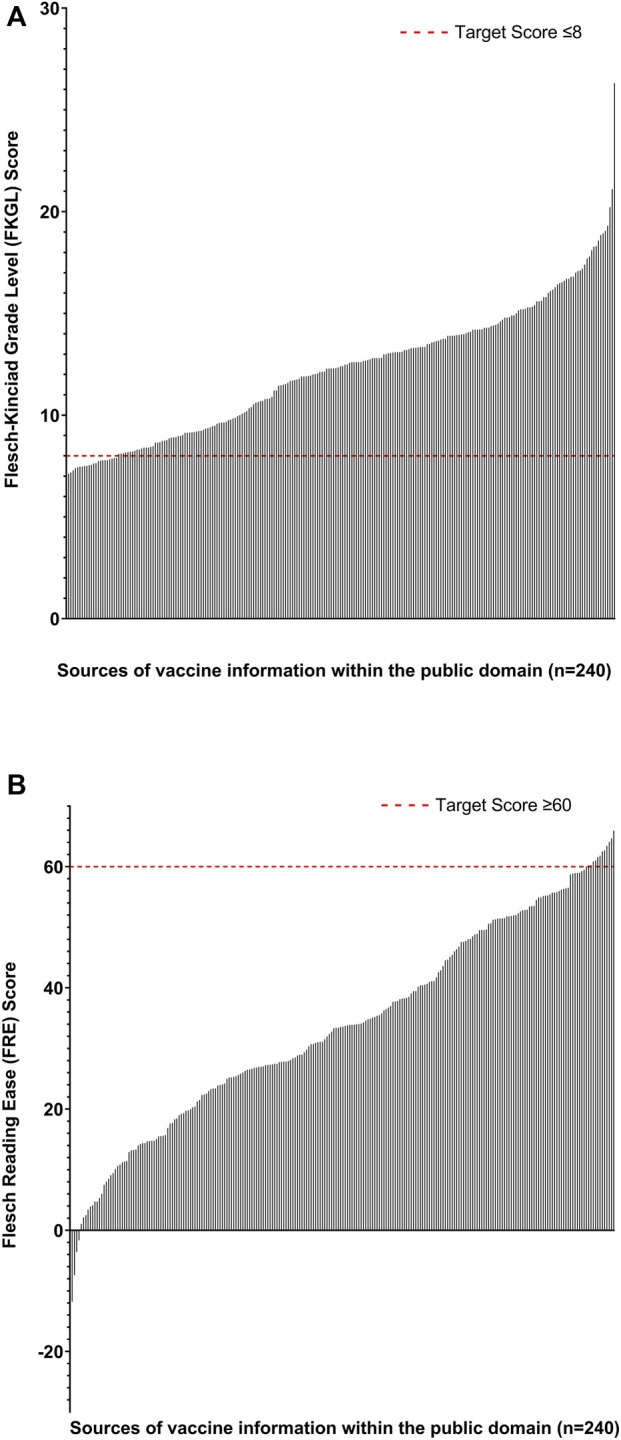
Analyses of readability scores for all retrieved sources of vaccine-related information (n = 240) which were within the public domain. **(A)** Flesch Kincaid Grade Level Score. The red line represents the target readability score based on US education grade levels (≤8). **(B)** Flesch Reading Ease Score. The red line represents the target score (≥60).

**FIGURE 3:**
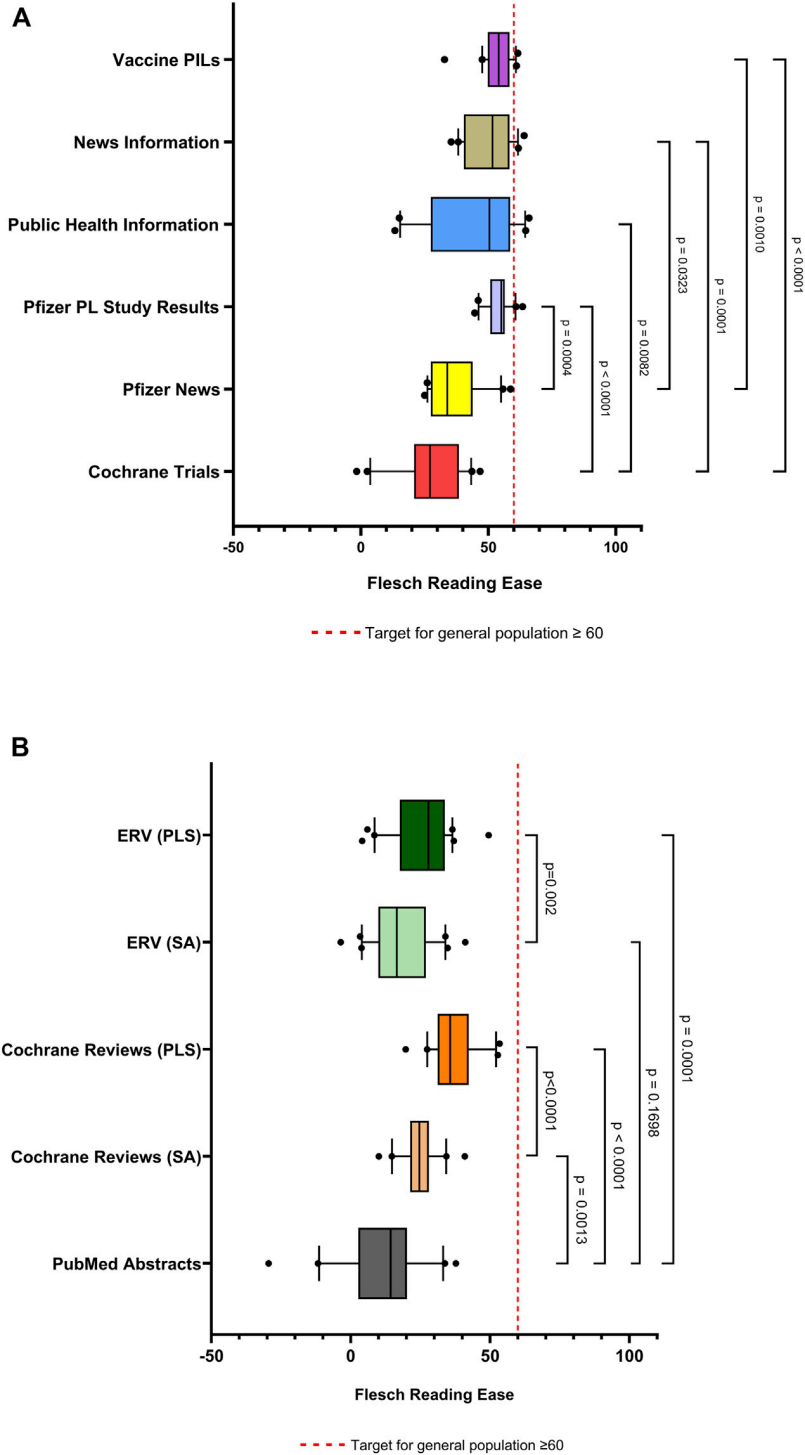
Box and whisker plots comparing Flesch Reading Ease scores of vaccine-related information. **(A)** Comparison of the readability of public facing lay information, comprising of information (n = 20 from each source) namely Cochrane Trials, Pfizer News, Pfizer plain language study results, public health information, news information and vaccine Patient Information Leaflets (PILs). **(B)** Comparison of the readability of public facing scientific information, namely PubMed abstracts (n = 20) and scientific abstracts and plain language summaries sourced from Cochrane Reviews (n = 20 paired) and Expert Review of Vaccines (n = 30 paired). Box represents 25th and 75th percentile and bar represents the median. Whiskers represent the 10th and 90th percentile and • represent outliers outside these percentile ranges. The dashed red line represents the target readability score (≥60). A p value of <0.05 (5%) was considered as statistically significant. Footnote: ERV-Expert Review of Vaccines; PL-Plain Language, PLS-Plain Language Summaries, PIL-Patient Information Leaflets, SA-Scientific Abstracts.

**FIGURE 4:**
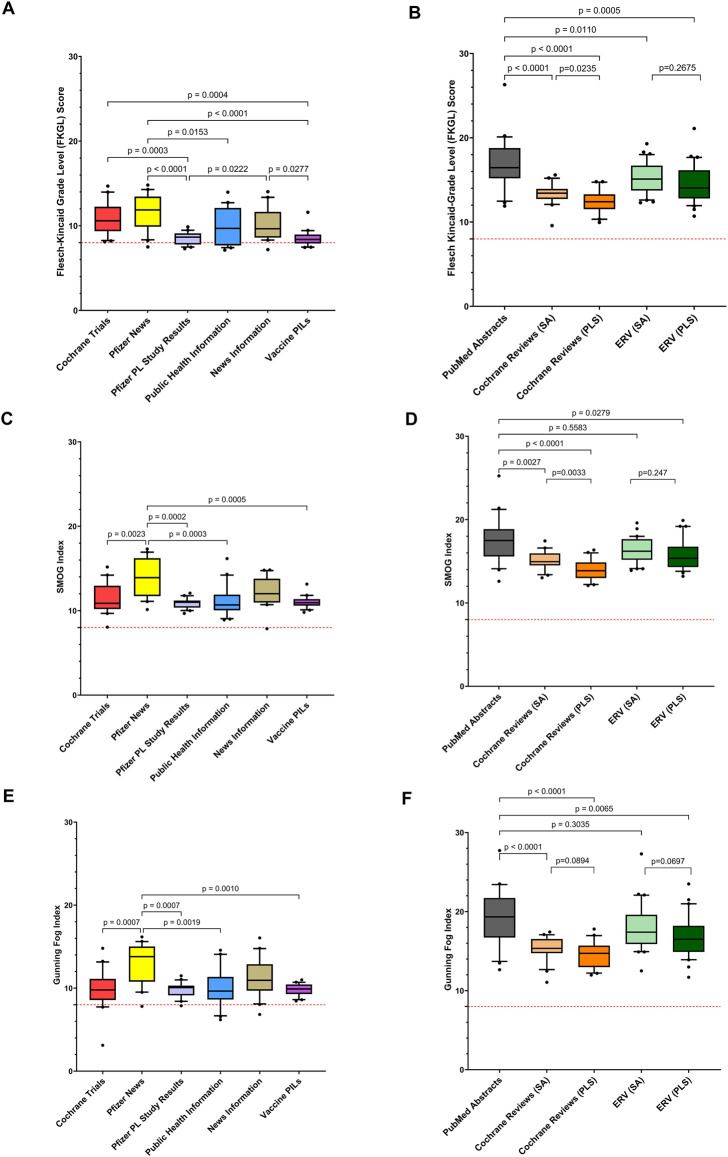
Box and whisker plots comparing readability scores; Flesch-Kincaid Grade Level **(A,B)**, SMOG Score **(C,D)** and Gunning Fog Score **(E,F)**; relating to public facing lay information (n = 20 from each source) **(A,C,E)** and publicly available scientific information, namely PubMed abstracts (n = 20) and scientific abstracts and plain language summaries sourced from Cochrane Reviews (n = 20 paired) and Expert Review of Vaccines (n = 30 paired). **(B,D,F)**. Box represents 25th and 75th percentile and bar represents the median. Whiskers represent the 10th and 90th percentile and • represent outliers outside these percentile ranges. The dashed red line represents the target readability score (≥8). A p value of <0.05 (5%) was considered as statistically significant. Footnote: ERV-Expert Review of Vaccines; PL-Plain Language, PLS-Plain Language Summaries, PIL-Patient Information Leaflets, SA-Scientific Abstracts.

**FIGURE 5:**
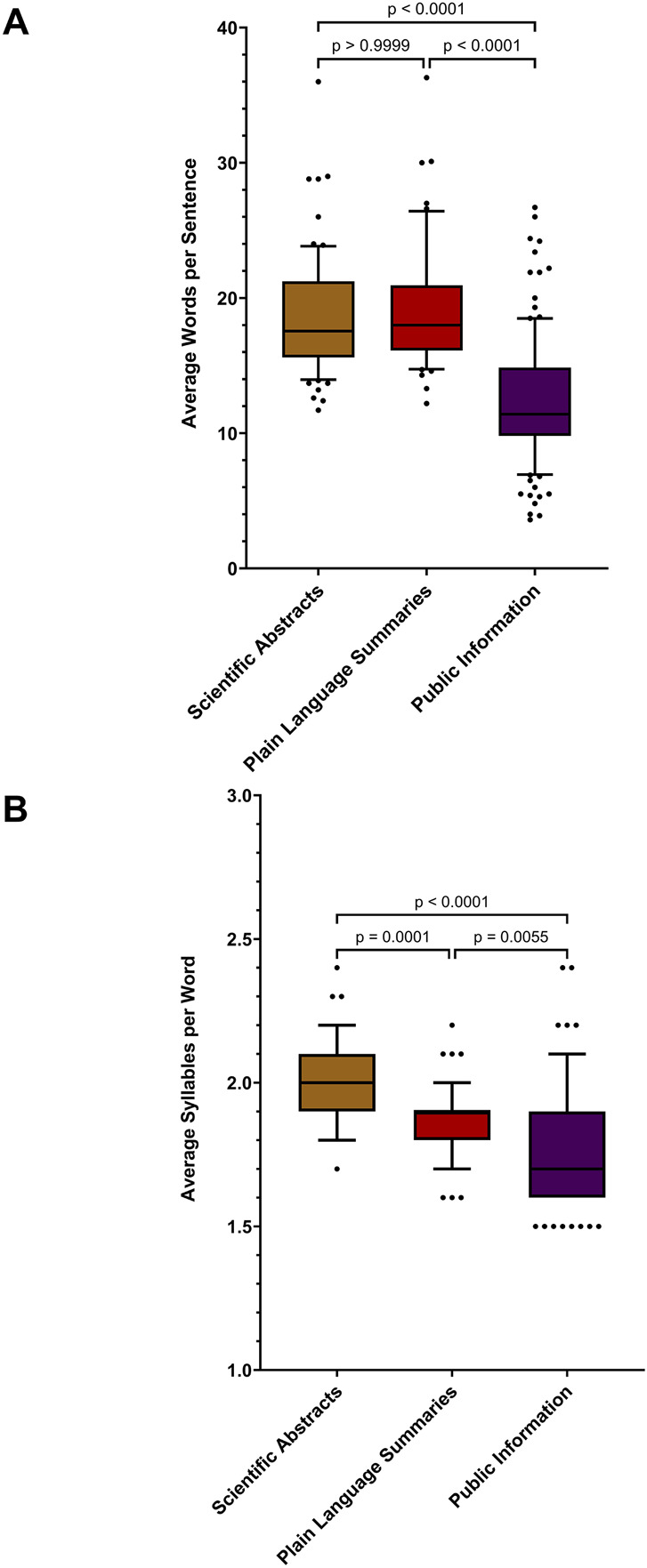
Box and whisker plots comparing: **(A)** Average Words per sentence; and **(B)** Average Syllables per word relating to three categories Scientific Abstracts (n = 70), Plain Language Summaries (n = 50) and Public Information bespoke for a lay audience (n = 120). Box represents 25th and 75th percentile and bar represents the median. Whiskers represent the 10th and 90th percentile and • represent outliers outside these percentile ranges. A p-value of <0.05 (5%) was considered as statistically significant.

All data collected relating to the four readability metrics and two sentence structure metrics are represented in Figures 2–5. Although all sources of information were free and in the public domain, for analyses and interpretation of the readability metrics, sources of information were divided into two main categories, namely (i) public facing, comprising of lay information sourced from Cochrane Trials, Pfizer News, Pfizer plain language study results, public health information, news information and vaccine PILs and (ii) scientific information, which were available to the public both written for the scientific and lay communities, namely PubMed abstracts and scientific abstracts and plain language summaries sourced from *Cochrane Reviews* and *Expert Review of Vaccines*.

### Readability of Total Information Retrieved

Of the total 240 retrieved sources of vaccine-related information which were within the public domain, 90.8% and 94.6% had poor readability in that they did not reach the target FKGL (≤8) (mean 12 ± 3.2) and FRE (≥60) (34 ± 17), respectively (Figures 2A,B).

### Scores of Public Facing Materials

The readability of the individual sources of public facing online information is represented in Figures 2, 3A, 4A,C,E; Supplementary Table S1. A similar trend of not reaching the targets for the general population is noted in the case of all four readability metrics. In relation to the FKGL (Figure 4) and FRE (Figure 3), of note was the Pfizer plain language study, where results were the closest to the accepted public targets for the FKGL (≤8) and the FRE (≥60) (mean 8.5 ± 0.8 sd; median 8.7) and (mean 53.9 ± 5.0 sd; median 55.1), respectively and also that the range of readability scores within these Pfizer study results were the smallest, 2.6 and 18.76, respectively, possibly indicating that a structured template was used to prepare such written materials, with a limited number of authors of such materials (Figures 4A, 3A). The vaccine PILs FRE (mean 53.9 ± 5.0 sd; median 54.0) (Figure 3A) and FKGL; (mean 8.5 ± 0.9 sd; median 8.4) (Figure 4A) also approached the acceptable public targets and the smaller ranges of 4.2 and 28.8 for the FKGL and FRE, respectively, which can be contributed to the formal structure template that all pharmaceutical companies adhere to when preparing a PIL. In contrast, the public health information which was written by several different organisations had greater ranges in relation to the FKGL and FRE being 6.8 and 52.6, respectively (Figures 4A, 3A). Similar trends were noted with the SMOG Index and the Gunning FOG Index for all public facing materials (Figures 3, 4).

### Readability Scores of Scientific Information

Regarding the scientific information, it was noted that the PubMed abstracts FRE score (mean 12 ± 16 sd; median 14, minimum value of −29 and maximum value of 38), indicated the poor readability of this written text, which was also reflected in the FKGL (mean 17 ± 3.1 sd; median value of 16 and a minimum value of 12 and maximum value of 26). The range of values as indicated in all four readability metrics was the highest for all metrics, indicating the variability of the authors writing (Figures 3, 4; Supplementary Table S2).

On comparison of the scientific abstracts and paired PLSs for both the *Cochrane Reviews* (CR) and *Expert Review of Vaccines* (ERV), there was a statistical significant difference between the scientific and plain language readability scores for the CR FRE (mean 25 ± 7.2 sd; median 25) versus (mean 37 ± 8.6 sd; median 36) p < 0.0001) and ERV FRE (mean 18 ± 11; median 17) versus (mean 26 ± 11; median 28) p = 0.002), respectively, however the readability of the plain language summaries did not meet the target score of 60 for the general public, with similar trend noted for all readability metrics (Figures 3, 4; Supplementary Table S2).

### Sentence Structure of Public Facing and Scientific Sources of Information

For the purposes of guidance to authors, the sentence structure of all sources of information were examined and it was evidenced that there were statistical differences in relation to the average words per sentence and average syllables per word as noted in Figures 5A,B respectively. Scientific vaccine-related abstracts for analyses purposes, comprised of data collated from abstracts sourced from PubMed, *Cochrane Reviews* and *Expert Review of Vaccines* abstracts. Plain language summaries comprised of data sources from *Cochrane Reviews* and *Expert Review of Vaccines*.

With regard the median average words per sentence, there was no statistical difference between the scientific abstracts and the plain language summaries (18, 95%CI: 17–19 and 18, 95%CI: 18-19), p > 0.9999, respectively. In contrast the median average words per sentence in relation to public facing information (11, 95%CI: 11-12), was statistically (p < 0.001) lower than both the scientific and plain language summaries (Figure 5A).

Scientific abstracts had a statistically higher median relating to average syllables per word compared to research information written in plain language and public facing information (2.0, 95% CI:2.0–2.0), (1.9, 95% CI:1.8–1.9) and (1.7, 95% CI: 1.7–1.8), respectively (Figure 5B). The lower number of words per sentence and syllables per words of the public facing information is reflected in the readability scores which are more in line with the target public audience.

## Discussion

The current study examined the readability of online vaccine-related information from ten reputable categories of online resources (n = 220) prepared by healthcare professionals, public health/governmental bodies and scientific and clinical researchers, in addition to a further source of information prepared by journalists of news articles (n = 20). Target readability scores are not formalised globally, however, within the published literature, relating to the general public, scores reported are based on the US schooling system, with a target reading age target of between Grade 6 and Grade 8 and most published research studies setting a target of readability scores of ≤ Grade 8 and a FRE of ≥60 and as such these were the targets used in this study [20, 22, 23]. According to the NHS Health Literacy Toolkit, the school grade in the US is one less than the UK as such US Grade 6 is the same as UK year 7, or age 11-12 and US Grade 8, is the same as UK year 9 or 13-14 [24]. The FRE reading score represents an arbitrary value with 60–70 equivalent to a US Grade Level of 8–9 [9].

It must be noted however that such targets are variable dependent on country, organisation and the target audience. One of the key findings of this study is that regarding the 240 retrieved sources of vaccine-related information which were within the public domain, 90.8% and 94.6% had poor readability in that they did not reach the target FKGL and FRE respectively. The difficulties of preparing written healthcare information at a level targeting the general public is not unique to public health guidance relating to vaccines and vaccination. Similar studies have been performed in relation to many other disciplines within healthcare including internet sources of information for health conditions which are frequently searched by patients, including online resources for cancer [25], cardiovascular disease [26], open-angle glaucoma [27], chronic health conditions [28] and tuberculosis [21], all of which have reported FKGL grade levels above 9.

The findings of this current study concur with a recent systematic review which examined the peer-reviewed literature for vaccine information readability studies and found that such information was written at a level higher than eighth grade [29]. Okuhara et al, included only twelve studies published prior to September 2020, ten of which were published in the English language, and two in Japanese, regarding the readability assessment of vaccine information, furthermore ten of these articles were published since 2016 [29]. The studies included in the systematic review related to governmental medical information, online immunization resources, pro and anti-vaccination websites and Facebook postings and included information prepared by both healthcare professionals and non-healthcare professionals. As the majority of studies evaluated focused on Human Papilloma Virus (HPV) vaccination, the authors suggested that more research should be conducted regarding the readability of vaccine information for other infectious diseases. In contrast, this current study looked a broad range of vaccine information and did not focus on one particular vaccination programme and as such showed that the overall readability of on-line vaccine information is too difficult for the general population, irrespective of the vaccine or vaccination programme.

Subsequent to the systematic review by Okuhara et al. and the COVID-19 pandemic, several publications globally have examined the readability of COVID-19 vaccine information and reported such information had poor readability scores of approximately tenth grade or higher [16, 17], with one study quoting FKGL of official websites ranging from 6.5 to 17.6 and FRE scores between 11.2 and 69.5 [15]. On a positive note was the fact that in a previous study by our group that the readability of twenty three different Vaccine Information Statements sourced from the US Centers for Disease Control and Prevention and “*Vaccines by Disease*” information from the US Department of Health & Human Services (HHS) had an average FKGL of 8.7 and 6.9, and FRE of 53.0 and 63.9, respectively [30], indicating that it is possible to achieve readability targets, with vaccine information.

Today with the availability of artificial intelligence, chatbots and online resources, many individuals can be defined as “internet health information seeking” or “health surfer” individuals. The availability of online sources of healthcare-related information, provides an opportunity for the public to become more informed and influenced in relation to their behaviours and healthcare decisions [31]. There are advantages to this internet-based approach to healthcare, in that individuals can be more informed and take ownership of their healthcare choices. In contrast, misinformation can result in individuals who now feel empowered to challenge their healthcare provider due to the misinformation they have sourced [32]. As such, the onus is on healthcare professionals and researchers to seek opportunities to deliver reliable vaccine-related information in a format which targets the level of understanding for the lay audience. In turn, individuals, irrespective of their level of education and level of understanding, will use such robust information to make accurately informed healthcare decisions [31].

Of concern, are the results of a Japanese study which compared the readability of anti-versus pro-influenza vaccine messages, written by healthcare and non-healthcare professionals and delivered online, which showed that anti-vaccination messages were more readable than pro-messages prepared by both healthcare and non-healthcare professionals [33]. Although the readability of Japanese written text is analysed using different language specific readability tools than those used for English text, this highlights the fact that readability of vaccine information can be poor irrespective of the language used in the text and there is a requirement for the preparation of such written materials to be improved globally.

Vaccination hesitancy or resistance has been reported to be fuelled by the availability of internet websites, which contains both pro- and anti-vaccination information [34]. Generally pro-vaccination websites, which are provided by authoritative bodies, such as governmental or healthcare institutions and scientific researchers, do not provide the opportunity for interaction with the general public and as such the communication is unidirectional, reinforcing the stance of these providers. In contrast, the vaccine-sceptical websites provide opportunities for individuals to share vaccination experiences which have affected them and additionally provide a platform to challenge the information provided by authoritative bodies. As such, the internet has become populated with websites which comprise of user generated information, Web 2.0, which potentially can be persuasive in making choices against vaccination [34]. This further places an onus on healthcare providers to provide information which the various lay stakeholder groups seek and in a format which they find conveys information in an appropriate format to make informed choices. One recent innovative approach to involve patients in designing information which they required to make an informed decision, was a co-production initiative, involving the design of a wordless picture book for adults with intellectual disabilities regarding COVID-19 vaccination [35]. This recent study highlighted the role that different communication approaches such as visual communication can facilitate discussions, provide support, alleviate fears and enhance health outcomes, particularly at a time when governmental documents were difficult to translate into readable formats [35].

Another key outcome from this current study was that although authors of research articles moderated and improved on the readability of PLSs in comparison to the paired scientific articles, the readability of such articles was still difficult. Conventionally, scientists disseminate their research findings by publication in peer-reviewed scientific journals for a target audience of mainly other scientists, who are knowledgeable and working in a similar field [36]. Due to the primary target audience, the authors of such publications tend to use technical or scientific language specific to the specialism to communicate and interpret the significance of their work, which may result in a barrier in understanding for individuals outside of the specialism and members of the lay community [36]. To address this issue various alternative forms of communication should be considered such as plain language summaries, podcasts, videos, picture infographics and the involvement of lay stakeholders in designing such communication materials. As highlighted in Figure 1, there are many occasions where it is necessary for scientists to communicate their research findings to the lay community and some journals are now providing welcomed opportunities to do so by inviting authors of scientific articles to publish a PLS alongside the scientific abstract of their full paper. A recent study which examined 534 health journals found only 27 (5.1%) provided an opportunity for a PLS and of those it was not a requirement for 70% of the journals [37].

To date there is a lack of consensus in the terminology used to describe such written text, e.g. plain language summaries, lay abstracts, lay summaries, clinical trial results for lay persons etc., however all are written for a target lay audience [36, 37]. Such lay summaries have various purposes including promotion as a visual presence, assisting in patient decision making, promoting public engagement in scientific research and sharing results of clinical trials for which lay communities have played a participating role or to encourage research collaboration. Furthermore, the readability target is dependent on the target audience which is a result of the purpose of the plain language summary, as detailed above.

Although PLSs are being increasingly introduced into scientific journals, a study by Zarshenas et al. reported that the guidance provided to prepare such summaries is generally limited to not using scientific jargon and complex words, and currently there are limited opportunities to provide patient and public partnerships to ensure that the outputs of such lay writing are optimal, which needs to be addressed [38]. A key factor which may greatly impact the readability of a PLS is the varied formats that the journals set in relation to the number of sentences, number of characters and structure, and specified content; however the instructions provided to authors are varied and guidance on readability levels and tools not routinely provided or differing [37, 38]. Although guidance relating to reading grades have been historically provided by various healthcare institutions such as the National Health Service (NHS) in the UK, the National Institutes of Health USA, American Medical Association and Centers for Disease Control and Prevention (CDC), a target of between Grade 6–8 has been commonly cited in the scientific literature [24, 39], it is important to consider the preferences of readers of PLSs. A recent study concluded a preference by participants of a medium-complexity, equating to a reading age of 14–17 years i.e. US Grade 9–11, however this study may not be representative of the general public as 90% of study participants were female, information seeking-individuals and 46%–50% were educated to degree level [40]. It is therefore important that future studies examine the readability preferences for a more representative lay reader cohort to determine target audience preferences to help ensure that the appropriate reading level is considered whilst not being too simplistic but remaining inclusive. It has been previously shown, that when the primary aim of a PLS is to increase knowledge, scientists summarising their research should consider the fact that lay audiences prefer structured formats with headings, the inclusion of background information, concluding statements and accompanying numerical information in the form of tables [36]. Sub-headings have been attributed to have many advantages over those without from a lay perspective relating to outcomes, such as confirmation of the credibility and accuracy of evidence and promotion of empowerment to make evidence-based decisions [36].

A recent UK study by Chisnall and colleagues, investigated the barriers to the uptake of childhood vaccines and the primary theme observed related to the provision of information both prior to and during the vaccination clinic [4]. Indeed, the lack of information resulted in parents searching for information on the internet or social media, which potentially had negative outcomes in deferring or refusing vaccination [4]. Parents indicated that they would have welcomed the opportunity to discuss vaccination to address any concerns or questions they had both prior to and during the vaccination clinic [4]. Parents also highlighted that they would have welcomed the availability of written information in an appropriate timeline to aid in the decision-making process [4]. It is noteworthy that when such information is not provided, patients preferably sourced relevant information from reputable online resources such as the NHS. This was due to the fact that misinformation materials online are not particularly evident and are well written, therefore making it difficult to assess what is valid information [4].

Occupational vaccination of healthcare workers including those working in hospital laboratories and handling cultures of pathogens or biological specimens is another important aspect of vaccine awareness. The literature has reported that vaccination coverage is not optimal in the healthcare cohort [41] and such suboptimal coverage poses risks not only to staff but also impacts on patient safety, particularly in the case of vulnerable patients [42]. The education of healthcare staff has been proposed to aid in occupational vaccination uptake. Future studies may consider investigating the role of readability of vaccine materials and the rate of vaccine uptake amongst healthcare workers.

Healthcare professionals, allied healthcare professionals and public health providers have a central role to promote vaccination awareness to various stakeholders including service users and healthcare/allied healthcare colleagues. The Health and Care Professions Council (HCPC) Standards of Proficiency outline the competencies of biomedical scientists and clinical scientists in terms of the promotion of health and the prevention of ill-health (Standard 15) and effective communication (Standard 7) and it is important to highlight the essential role that registrants can play in the promotion and dissemination of vaccination awareness in-line with these standards (see Table 2) [43, 44].

**TABLE 2:** The Health & Care Professions Council Standards of Proficiency relevant to the communication of information related to vaccination. © The Health and Care Professions Council (1 September 2023).

Standard 7	Communicate effectively
7.1 (BMS and CS)	Use effective and appropriate verbal and non-verbal skills to communicate with service users, carers, colleagues and others
7.3 (BMS and CS)	Understand the characteristics and consequences of verbal and non-verbal communication and recognise how these can be affected by difference of any kind, including, but not limited to, protected characteristics^a^, intersectional experiences and cultural differences
7.4 (BMS and CS)	Work with service users and/or their carers to facilitate the service user’s preferred role in decision-making, and provide service users and carers with the information they may need where appropriate
7.5 (BMS and CS)	Modify their own means of communication to address the individual communication needs and preferences of service users and carers, and remove any barriers to communication where possible
7.6 (BMS and CS)	Understand the need to support the communication needs of service users and carers, such as through the use of an appropriate interpreter
7.7 (BMS and CS)	Use information, communication and digital technologies appropriate to their practice
7.8 (BMS and CS)	Understand the need to provide service users or people acting on their behalf with the information necessary, in accessible formats, to enable them to make informed decisions
7.9 (CS)	Communicate the outcome of problem-solving and research and developmental activities
7.10 (CS)	Summarise and present complex scientific ideas in an appropriate form

Standard 15	Promote health and prevent ill health
15.1 (BMS and CS)	Understand the role of their profession in health promotion, health education and preventing ill health
15.2 (BMS and CS)	Understand how social, economic and environmental factors (wider determinants of health) can influence a person’s health and wellbeing
15.3 (BMS and CS)	Empower and enable individuals (including service users and colleagues) to play a part in managing their own health
15.4 (BMS and CS)	Engage in occupational health, including being aware of immunisation requirements

Standard 7 and Standard 15 taken from “Standards of proficiency – Biomedical scientists” and “Standards of proficiency – Clinical scientists” with permission from The Health and Care Professions Council (2023), www.hcpc-uk.org.

^a^ The Equality Act 2010 defines the protected characteristics as age, disability, gender reassignment, race, religion or belief, sex, sexual orientation, marriage and civil partnership and pregnancy and maternity. Equivalent equality legislation in Northern Ireland protects age, disability, gender, race, religion or belief and sexual orientation.

Abbreviations: BMS, biomedical scientists; CS, clinical scientists.

**FIGURE 6:**
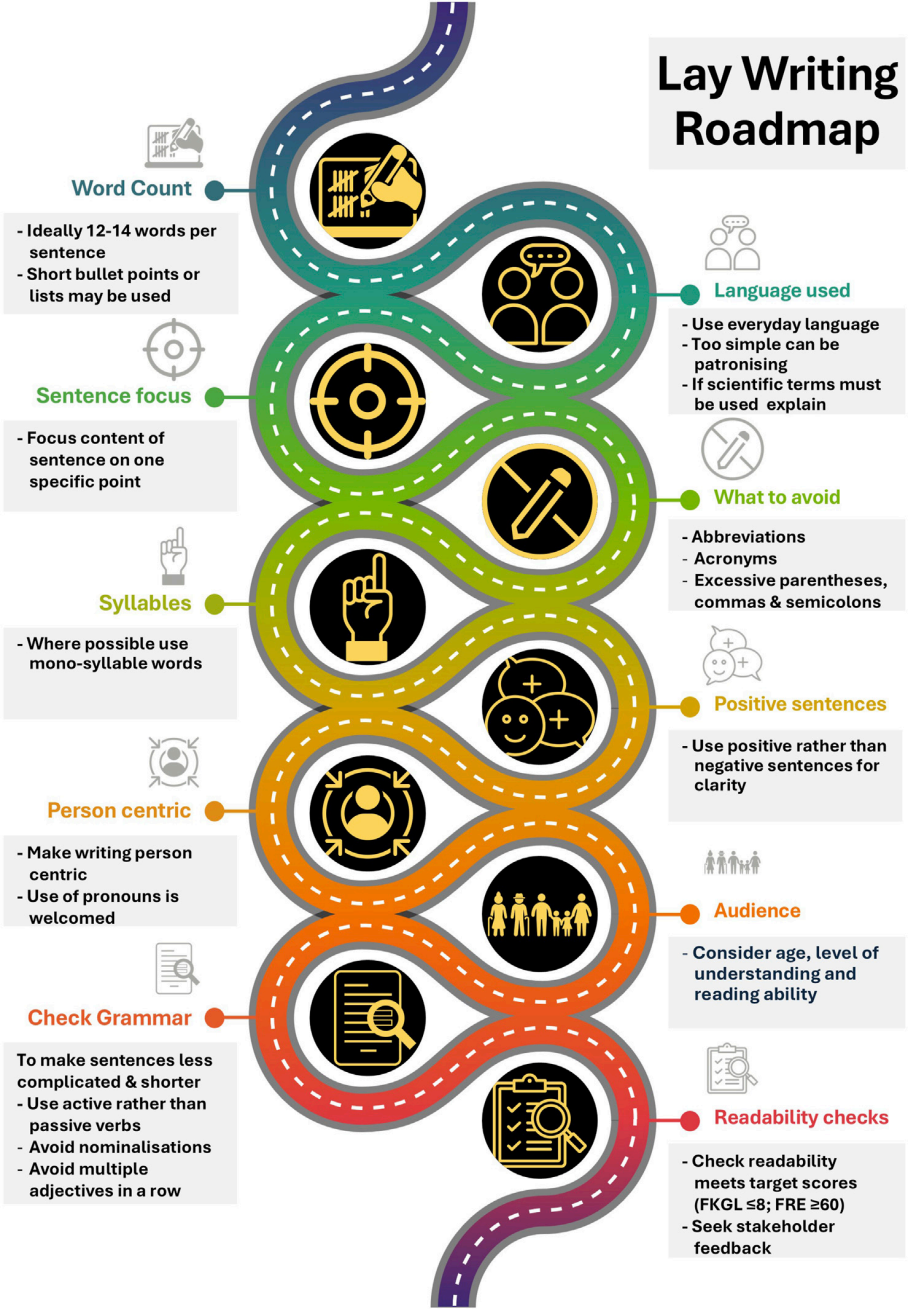
Guidance relating to the preparation of lay materials to help ensure that the readability targets for the public are achieved. Footnote: FKGL, Flesch-Kincaid Grade Level; FRE, Flesch Reading Ease.

Figure 6 details key points to consider when preparing such written lay materials. Further valuable resources are available from Taylor and Francis [14], Elsevier [45] and Cochrane [46] as well as healthcare/public health and governmental bodies such as the CDC [47], National Institute of Health [48] and NHS [24] and Orritt & Powell’s article on how to talk to the public [49].

Some limitations of this current study are acknowledged. Readability tools are based on using formulae calculations to assess how difficult a piece of text is to read and are an important initial step in examining materials for a lay audience, however such metrics, do not consider the accuracy of the written content and as such it is the responsibility of the healthcare professional or healthcare scientist to ensure that all information is based on robust evidence-based research. Additionally, readability scores do not directly assess the target audience’s level of comprehension. Factors other than sentence structure can impact on the target audience’s ability to comprehend the information provided, such as visual attributes including font size, colour schemes, spacing, icons, infographics, visuals and overall layout. In addition, current readability scales, including the ones used in this study, do not adequately measure visual aids effectively [8, 50], which are relevant to enhance health literacy in the case of individuals with low-literary skills [51]. Furthermore, such representations have been shown to be preferred by the lay community [40] and are often used in conjunction with text, or to supplement it, as such, readability scores may not accurately reflect the overall readability of a text, despite being understandable by the majority of the population. It is therefore important to collaborate with the intended audience to co-produce and pre-test written materials containing the key relevant and evidence-based information, prior to rollout of such materials [24]. In the preparation of vaccine information, it is important to centralise the recipient or in the case of the child also their parents. As such going forward, stakeholder focus groups should consider a partnership involvement in development of such materials and incorporate the key information and concerns that stakeholders require to make informed decisions as well as the optimum communication approach, whether that be verbal, visual or written.

## Conclusion

Overall, the readability of public facing online resources relating to vaccines does not meet recommended reading grade levels and as such could be improved by considering the use of readability tools to help prepare lay materials which are easier to read. It is important for authors to have and follow clear guidelines to lay writing. It is fundamentally important that when preparing public facing materials, scientists and healthcare professionals should consider patient public partnerships to prepare materials which are readable, comprehensive and purposeful.

## Summary Table

### What Is Known About This Topic

- The uptake of vaccines has decreased resulting in increasing incidence and re-emergence of infectious diseases
- In fulfilment of HCPC Standards of Proficiency, Biomedical scientists (BMSs) have a role in health promotion and prevention of ill health.
- BMSs must communicate effectively with service users to make informed decisions.

### What This Work Adds

- The readability of vaccine information is poor and does not reach the readability standards for the general public.
- The BMS community should provide vaccine information to stakeholders, service users and lay community in an appropriate format to facilitate evidence-based decisions
- Vaccine information should be prepared and checked to ensure its readability meets international targets of FKGL of Target ≤8th Grade.

## Concluding Statement

This work represents an advance in biomedical science as analysis of vaccine information which is available to the public has indicated poor readability scores. To ensure that the biomedical scientist community provides information in a more readable format such information should be checked using readability calculators prior to distribution. This is one aspect which could enhance the promotion of vaccine awareness in an attempt to improve vaccination uptake rates.

## Data Availability

The original contributions presented in the study are included in the article/Supplementary Material, further inquiries can be directed to the corresponding author.
