# Supplementary material for: An Analysis of the Readability of Public-Facing Information Relating to Prevention of Infectious Diseases by Vaccination
Source: Br J Biomed Sci. 2025 Dec 22;82:15435. doi: 10.3389/bjbs.2025.15435 (PMC12766163; doi:10.3389/bjbs.2025.15435)
Supplement: Supplementary file 1 [file DataSheet2.pdf]

Table 2: Descriptive statistics of readability scores relating to scientific vaccination information

A: Flesch Reading Ease (FRE)	Cochrane Reviews (SA)	Cochrane Reviews (PLS)	PubMed (SA)	ERV (SA)	ERV (PLS)
Number of values	20	20	20	30	30
Minimum	10	20	-29	-3.6	4.1
25% Percentile	21	31	2.8	10	18
Median	25	36	14	17	28
75% Percentile	28	42	20	27	34
Maximum	41	53	38	41	50
Range	31	34	67	45	45
95% CI of median					
Actual confidence level	96%	96%	96%	96%	96%
Lower confidence limit	21	32	4.7	11	23
Upper confidence limit	28	41	20	24	32
Mean	25	37	12	18	26
Std. Deviation	7.2	8.6	16	11	11
Std. Error of Mean	1.6	1.9	3.5	2.0	2.0
Lower 95% CI of mean	21	33	4.5	14	22
Upper 95% CI of mean	28	41	19	22	30
Coefficient of variation	29%	23%	132%	62%	42%

B: Flesch-Kincaid Grade Level (FKGL)	Cochrane Reviews (SA)	Cochrane Reviews (PLS)	PubMed (SA)	ERV (SA)	ERV (PLS)
Number of values	20	20	20	30	30
Minimum	9.6	10	12	12	11
25% Percentile	13	12	15	14	13
Median	13	12	16	15	14
75% Percentile	14	13	19	17	16
Maximum	16	15	26	19	21
Range	6.0	4.8	14	7.0	10
95% CI of median					
Actual confidence level	96%	96%	96%	96%	96%
Lower confidence limit	13	12	15	14	13
Upper confidence limit	14	13	19	16	15
Mean	13	12	17	15	14
Std. Deviation	1.3	1.4	3.1	1.9	2.3
Std. Error of Mean	0.29	0.31	0.70	0.34	0.42
Lower 95% CI of mean	13	12	15	14	14
Upper 95% CI of mean	14	13	18	16	15
Coefficient of variation	9.6%	11%	19%	12%	16%

C: SMOG	Cochrane Reviews (SA)	Cochrane Reviews (PLS)	PubMed (SA)	ERV (SA)	ERV (PLS)
Number of values	20	20	20	30	30
Minimum	13.0	12.1	12.6	13.9	13.2
25% Percentile	14.5	13.0	15.6	15.2	14.3
Median	14.9	13.9	17.5	16.2	15.4
75% Percentile	16.0	14.9	18.9	17.7	16.8
Maximum	17.4	16.4	25.3	19.6	19.9
Range	4.42	4.26	12.7	5.70	6.70
95% CI of median					
Actual confidence level	95.9%	95.9%	95.9%	95.7%	95.7%
Lower confidence limit	14.5	13.2	15.9	15.5	14.7
Upper confidence limit	15.8	14.6	18.8	16.9	15.9
Mean	15.1	14.0	17.6	16.2	15.8
Std. Deviation	1.14	1.21	2.75	1.49	1.84
Std. Error of Mean	0.256	0.270	0.615	0.271	0.337
Lower 95% CI of mean	14.6	13.4	16.4	15.7	15.1
Upper 95% CI of mean	15.6	14.5	18.9	16.8	16.5
Coefficient of variation	7.57%	8.63%	15.6%	9.15%	11.7%

D: Gunning Fog	Cochrane Reviews (SA)	Cochrane Reviews (PLS)	PubMed (SA)	ERV (SA)	ERV (PLS)
Number of values	20	20	20	30	30
Minimum	11.1	12.0	12.7	12.5	11.7
25% Percentile	14.7	13.0	16.7	15.9	14.9
Median	15.3	14.7	19.3	17.4	16.5
75% Percentile	16.5	15.7	21.7	19.6	18.2
Maximum	17.4	17.8	27.7	27.3	23.5
Range	6.36	5.82	15.1	14.8	11.8
95% CI of median					
Actual confidence level	95.9%	95.9%	95.9%	95.7%	95.7%
Lower confidence limit	15.0	13.3	17.6	16.5	15.1
Upper confidence limit	16.3	15.3	21.5	19.0	17.4
Mean	15.4	14.5	19.2	18.0	16.8
Std. Deviation	1.53	1.65	3.52	2.89	2.71
Std. Error of Mean	0.342	0.368	0.788	0.528	0.496
Lower 95% CI of mean	14.6	13.7	17.6	16.9	15.8
Upper 95% CI of mean	16.1	15.2	20.9	19.1	17.8
Coefficient of variation	9.96%	11.4%	18.4%	16.1%	16.2%

Abbreviations: PLs, Plain Language Summary; SA, Scientific Abstract; SMOG, Simple Measure of Gobbledygook
