# Supplementary material for: An Analysis of the Readability of Public-Facing Information Relating to Prevention of Infectious Diseases by Vaccination
Source: Br J Biomed Sci. 2025 Dec 22;82:15435. doi: 10.3389/bjbs.2025.15435 (PMC12766163; doi:10.3389/bjbs.2025.15435)
Supplement: Supplementary file 2 [file DataSheet1.pdf]

Table 1: Descriptive statistics of readability scores relating to public facing vaccination information

A: Flesch Reading Ease (FRE)	Cochrane Trials	Pfizer News	Pfizer PL Study Results	Public Health Information	News Information	Vaccine PILs
Number of values	20	20	20	20	20	20
Minimum	-1.660	24.97	44.66	13.33	35.39	32.78
25% Percentile	20.88	27.49	50.75	27.48	40.41	49.82
Median	27.12	33.93	55.05	50.38	51.61	54.00
75% Percentile	38.45	43.85	56.41	58.58	58.30	58.26
Maximum	46.79	58.70	63.42	65.93	64.06	61.58
Range	48.45	33.73	18.76	52.60	28.67	28.80
95% CI of median						
Actual confidence level	95.86%	95.86%	95.86%	95.86%	95.86%	95.86%
Lower confidence limit	22.30	27.84	51.30	28.83	41.11	50.68
Upper confidence limit	38.24	41.71	56.35	53.52	55.44	56.50
Mean	27.44	36.75	53.93	43.71	49.65	53.15
Std. Deviation	12.87	10.10	4.976	17.29	9.027	6.403
Std. Error of Mean	2.879	2.259	1.113	3.867	2.018	1.432
Lower 95% CI of mean	21.42	32.02	51.60	35.61	45.42	50.15
Upper 95% CI of mean	33.47	41.48	56.26	51.80	53.87	56.14
Coefficient of variation	46.91%	27.49%	9.228%	39.57%	18.18%	12.05%

B: Flesch-Kincaid Grade Level (FKGL)	Cochrane Trials	Pfizer News	Pfizer PL Study Results	Public Health Information	News Information	Vaccine PILs
Number of values	20	20	20	20	20	20
Minimum	8.1	7.5	7.3	7.1	7.2	7.4
25% Percentile	9.4	9.9	7.8	7.7	8.6	7.9
Median	11	12	8.7	9.7	9.7	8.4
75% Percentile	12	13	9.1	12	12	9.0
Maximum	15	15	9.9	14	14	12
Range	6.6	7.3	2.6	6.8	6.8	4.2
95% CI of median						
Actual confidence level	96%	96%	96%	96%	96%	96%
Lower confidence limit	9.6	10	7.8	7.8	9.0	7.9
Upper confidence limit	12	13	8.9	12	12	8.9
Mean	11	12	8.5	9.9	10	8.5
Std. Deviation	2.0	2.1	0.75	2.2	1.9	0.93
Std. Error of Mean	0.44	0.48	0.17	0.50	0.43	0.21
Lower 95% CI of mean	10	11	8.1	8.9	9.3	8.1
Upper 95% CI of mean	12	13	8.8	11	11	9.0
Coefficient of variation	18%	18%	8.8%	22%	19%	11%

C: SMOG	Cochrane Trials	Pfizer News	Pfizer PL Study Results	Public Health Information	News Information	Vaccine PILs
Number of values	20	20	20	20	20	20
Minimum	8.05	10.1	9.68	8.91	7.87	9.81
25% Percentile	10.2	11.7	10.4	10.1	11.0	10.6
Median	10.9	13.9	11.0	10.7	12.0	10.9
75% Percentile	13.0	16.2	11.2	11.9	13.8	11.4
Maximum	15.2	17.3	12.1	16.2	14.8	13.2
Range	7.13	7.17	2.40	7.26	6.96	3.34
95% CI of median						
Actual confidence level	95.9%	95.9%	95.9%	95.9%	95.9%	95.9%
Lower confidence limit	10.4	12.0	10.6	10.1	11.0	10.6
Upper confidence limit	12.0	15.9	11.2	11.9	13.8	11.3
Mean	11.4	14.0	10.9	11.2	12.2	11.0
Std. Deviation	1.83	2.26	0.612	1.83	1.73	0.723
Std. Error of Mean	0.410	0.505	0.137	0.408	0.387	0.162
Lower 95% CI of mean	10.6	12.9	10.6	10.3	11.4	10.7
Upper 95% CI of mean	12.3	15.1	11.2	12.1	13.0	11.3
Coefficient of variation	16.1%	16.2%	5.62%	16.3%	14.2%	6.57%

D: Gunning Fog	Cochrane Trials	Pfizer News	Pfizer PL Study Results	Public Health Information	News Information	Vaccine PILs
**Number of values**	20	20	20	20	20	20
**Minimum**	3.12	7.80	7.87	6.20	6.83	8.45
**25% Percentile**	8.57	10.8	9.14	8.63	9.70	9.29
**Median**	9.79	13.8	10.1	9.65	10.9	9.90
**75% Percentile**	11.1	15.0	10.3	11.3	12.9	10.4
**Maximum**	14.8	16.2	11.5	14.6	16.1	11.0
**Range**	11.7	8.37	3.62	8.37	9.22	2.55
**95% CI of median**						
**Actual confidence level**	95.9%	95.9%	95.9%	95.9%	95.9%	95.9%
**Lower confidence limit**	8.78	11.5	9.23	9.15	10.1	9.30
**Upper confidence limit**	10.3	14.9	10.3	11.2	12.7	10.4
**Mean**	9.81	13.0	9.77	10.1	11.3	9.80
**Std. Deviation**	2.38	2.46	0.910	2.29	2.39	0.733
**Std. Error of Mean**	0.532	0.551	0.203	0.513	0.535	0.164
**Lower 95% CI of mean**	8.70	11.8	9.34	9.02	10.2	9.46
**Upper 95% CI of mean**	10.9	14.1	10.2	11.2	12.5	10.1
**Coefficient of variation**	24.3%	19.0%	9.32%	22.7%	21.1%	7.48%

Abbreviations: PL, Plain Language; PIL, Patient Information Leaflet; SMOG, Simple Measure of Gobbledygook
